# Transcriptional activation of *AaAGPase* by AaGLK transcription factor determines ascorbic acid levels in kiwifruit

**DOI:** 10.1093/hr/uhag212

**Published:** 2026-06-01

**Authors:** Sikai Li, Ran Wang, Miaomiao Lin, Leiming Sun, Yukuo Li, Li Liu, Ziqi Zhang, Xiujuan Qi

**Affiliations:** National Key Laboratory for Germplasm Innovation & Utilization of Horticultural Crops, Zhengzhou Fruit Research Institute, Chinese Academy of Agricultural Sciences, Zhengzhou 450009, Henan, China; National Key Laboratory for Germplasm Innovation & Utilization of Horticultural Crops, Zhengzhou Fruit Research Institute, Chinese Academy of Agricultural Sciences, Zhengzhou 450009, Henan, China; National Key Laboratory for Germplasm Innovation & Utilization of Horticultural Crops, Zhengzhou Fruit Research Institute, Chinese Academy of Agricultural Sciences, Zhengzhou 450009, Henan, China; National Key Laboratory for Germplasm Innovation & Utilization of Horticultural Crops, Zhengzhou Fruit Research Institute, Chinese Academy of Agricultural Sciences, Zhengzhou 450009, Henan, China; National Key Laboratory for Germplasm Innovation & Utilization of Horticultural Crops, Zhengzhou Fruit Research Institute, Chinese Academy of Agricultural Sciences, Zhengzhou 450009, Henan, China; College of Horticulture and Plant Protection, Henan University of Science and Technology,, Luoyang 471003, Henan, China; National Key Laboratory for Germplasm Innovation & Utilization of Horticultural Crops, Zhengzhou Fruit Research Institute, Chinese Academy of Agricultural Sciences, Zhengzhou 450009, Henan, China; National Key Laboratory for Germplasm Innovation & Utilization of Horticultural Crops, Zhengzhou Fruit Research Institute, Chinese Academy of Agricultural Sciences, Zhengzhou 450009, Henan, China

## Abstract

Humans cannot synthesize ascorbic acid (AsA), making dietary intake from fruits and vegetables crucial. Kiwifruit is a rich source of AsA; however, the genetic and regulatory basis for its accumulation is not fully elucidated. This study integrates quantitative trait locus (QTL) mapping, transcriptomics, and functional characterization to uncover a novel regulatory role of *AaAGPase*, encoding an ADP-glucose pyrophosphorylase, in determining AsA levels in kiwifruit. A significant QTL was mapped on chr10 in a tetraploid *Actinidia arguta* biparental population, explaining 11.46% of the variation in AsA levels. Based on gene annotation and expression differences between high- and low-AsA genotypes, ADP-glucose pyrophosphorylase (AGPase) was identified as a negative regulator. Our results indicated that a promoter variation in *AGPase* affects both AsA and starch levels. Overexpression of *AaAGPase* in transiently transformed fruit and transgenic kiwifruit plants led to reduced AsA levels via downregulation of key genes in the L-galactose pathway while increasing starch levels. Conversely, virus-induced gene silencing of *AaAGPase* resulted in increased AsA but decreased starch levels. Additionally, we identified the Golden 2-like transcription factor, AaGLK, which binds to and activates the *AaAGPase* promoter. Manipulating AaGLK expression resulted in variations in AsA levels by regulating *AaAGPase* expression. These findings reveal that the AaGLK-AaAGPase module negatively regulates AsA biosynthesis in kiwifruit. Additionally, we developed a molecular marker based on the sequence variation of *AGPase* promoter for marker assisted selection. In summary, our study revealed that *AaAGPase*, which is transcriptionally activated by AaGLK, plays a novel role in determining AsA levels through carbohydrates substrates competition.

## Introduction

L-ascorbic acid (AsA), also known as vitamin C, is an essential water-soluble antioxidant, and AsA plays crucial role in human health. It contributes to the maintenance of human cartilage and bones, the defense of the immune system and the prevention of cardiovascular diseases [1–3]. However, humans lack the ability to synthesize AsA due to mutations of key enzyme genes. Therefore, it must be supplied from dietary sources, mainly from fresh fruits and vegetables [4, 5]. Beyond its nutritional value, AsA plays crucial role in multiple biological processes in plants, such as photosynthesis, growth regulation, hormone signaling, and stress responses [3, 6–10].

In plants, AsA homeostasis is governed by the balance of biosynthesis, degradation, and recycling [2, 3, 11]. Four main biosynthetic pathways for AsA synthesis have been identified in plants: L-galactose, D-galacturonate, myo-inositol, and L-gulose [12–15]. The L-galactose pathway, also known as the Smirnoff–Wheeler pathway, is the main pathway for AsA biosynthesis with four key rate-limiting enzyme genes: mannose-1-phosphate guanylyltransferase (*GMP*), GDP-D-mannose-3′,5′-epimerase (*GME*), GDP-L-galactose phosphorylase (*GGP*), and L-galactose-1-phosphate phosphatase (*GPP*) [16–19]. The D-galacturonic acid pathway supports the L-galactose pathway, with GalUR as a key enzyme [13, 20]. Research on the inositol and L-gulose pathways is limited to model plants, while L-galactose dehydrogenase (GDH) and L-galactono-1,4-lactone dehydrogenase are essential for all four pathways [20, 21].

Kiwifruit (*Actinidia* spp.) is a dioecious, deciduous vine native to China. It is well known for its high AsA content and thus serves as an ideal model for investigating the mechanisms underlying AsA accumulation in fruit [22]. The *Actinidia* genus includes 54 species and 21 varieties, with high morphological diversity [23]. *Actinidia arguta* is the most widely distributed species within the genus [24]. Compared to the traditional brown, fuzzy kiwifruit, *A. arguta* fruit is smaller, similar to grape size, and has a smooth and edible skin [25]. Studies have indicated that the AsA content is higher in the skin of *A. arguta* fruit than in the flesh [26]. Most cultivated *A. arguta* varieties are polyploid, and unlike in diploid species, genetic research in polyploids faces significant challenges due to multiple sets of chromosomes. Trait segregation is highly complex and unpredictable, making it difficult to trace desired traits. In addition, kiwifruit has a long juvenile period, and traditional breeding strategies that rely on direct phenotypic selection are time-consuming. Molecular marker-assisted selection (MAS) offers an efficient alternative for developing new kiwifruit cultivars.

AsA biosynthesis in kiwifruit is mainly regulated by the L-galactose pathway, which is modulated by various hormone-responsive transcription factors [27]. Additionally, factors, such as recycling and degradation enzymes, genotype, developmental stages, and postharvest treatments, play essential roles in fine-tuning AsA content. The L-galactose pathway is crucial for AsA accumulation, while D-galacturonic acid and recycling pathways play secondary roles. Developmental profiling shows AsA peaks in early fruit development, before declining, closely mirroring the expression and activity of key L-galactose pathway enzymes, such as GME, GGP, GalDH, and GalLDH [27, 28]. GGP, particularly GGP3, is identified as the main rate-limiting step. A strong correlation exists between higher *GGP3* expression and increased AsA levels across various *Actinidia* species. Furthermore, the overexpression of *GGP3* in kiwifruit, Arabidopsis, tomato, and other plants results in a 2- to 7-fold increase in AsA [17, 28, 29]. The R1-type MYB AceMYBS1 is a direct activator: its overexpression increases *GGP3* transcription and raises AsA, while abscisic acid suppresses MYBS1, resulting in decreased AsA [3, 29]. The basic leucine Zipper (bZIP) factor AceGBF3 interacts with MYBS1 and together, they co-activate *GGP3*, further elevating AsA levels [3]. In contrast, the bZIP repressor AcABI5a inhibits both *MYBS1* expression and *GGP3* promoter activity, leading to reduced AsA levels when overexpressed, while its knockout produces the opposite effect [29]. Additionally, the cold-inducible bZIP factor AcePosF21 binds the *GGP3* promoter; its loss results in decreased AsA levels and increased reactive oxygen species accumulation [30]. The ethylene-responsive factor AcERF91 also activates *GGP3*, increasing AsA when overexpressed [31]. Although significant progress has been made in understanding the AsA biosynthesis in kiwifruit, the genetic basis of AsA biosynthesis is still limited.

The ADP-glucose pyrophosphorylase (AGPase) is crucial in the metabolic conversion of D-glucose-1-phosphate (D-glucose-1P) to ADP-glucose, providing the activated glucosyl donor required for starch synthesis in plants. As the first committed step in this pathway, AGPase serves as the primary regulatory gate that controls the flow of carbon into storage polysaccharides [32, 33]. AGPase is a heterotetramer of a small subunit and a large subunit, regulated by allosteric effectors such as 3-phosphoglycerate and inorganic phosphate, along with redox and other posttranslational modifications [34, 35]. Starch is a major component of seeds, tubers, and other storage tissues; thus, AGPase is a major target for crop engineering to modify starch yield and composition [33, 35]. In rice, a modified AGPase was introduced into seeds to express the enzyme in the cytoplasm or amyloplast of endosperm. This manipulation enhanced starch synthesis and seed weight by increasing cytoplasmic AGPase catalytic activity in transgenic rice [33].

Golden 2-like, also known as G2-like or GLK, was first identified in maize as a transcriptional regulator involved in maize leaf cell differentiation [36]. GLK transcription factors belong to the GARP family of MYB-related proteins and play critical roles in regulating chloroplast development and photosynthetic capacity in plants [37]. In *Arabidopsis thaliana*, two homologous genes, *GLK1* and *GLK2*, function redundantly to promote chloroplast biogenesis by activating the expression of nuclear genes associated with photosynthesis, chlorophyll biosynthesis, and light-harvesting complexes [38]. In apple, the GLK family gene *MdGLK1* can regulate the accumulation of chlorophyll and affect the development of chloroplasts [37]. In kiwifruit, a GLK homolog (*AchGLK*) has been isolated and functionally characterized [39]. These results indicate that GLK transcription factors play conserved roles in plastid development and may serve as promising targets for improving photosynthesis and fruit quality in horticultural crops.

Despite the well-established role of *AaAGPase* in starch accumulation, its involvement in AsA biosynthesis has yet to be investigated. The current study identified a QTL on chr10 in an *A. arguta* biparental population, with RNA sequencing pinpointing *AaAGPase* as the candidate determining AsA levels. We further characterized the function of *AGPase*, hypothesizing that it plays a regulatory role in AsA biosynthesis through modulating the carbohydrates substrates used for starch synthesis. Furthermore, we identified a member of the Golden 2-like transcription factor family, AaGLK, which binds to the promoter of *AaAGPase* and activates its transcription. Transient and stable transformations in kiwifurit demonstrated that AaGLK-AaAGPase module functions as a shared transcriptional relay for the regulation of AsA biosynthesis, with *AaAGPase* being the dominant factor. This study provides new insights into the molecular mechanism of AsA biosynthesis in plants, particularly in kiwifruit.

## Results

### Integrated QTL mapping and RNA-seq pinpoints *AaAGPase* determining AsA levels

In this study, the average AsA content of the female parent ‘Ruby-3’ fruit for three years was 365.28 μg/l. Statistical analysis revealed significant variation in AsA content within the F1 population. In 2021, the AsA content ranged from 136.59 to 439.10 μg/l; in 2022, it was between 267.45 and 518.93 μg/l; and in 2023, it varied from 248.18 to 520.49 μg/l. Shapiro–Wilk tests confirmed normal distribution across all 3 years (*P* > 0.05). This variability underscores the population’s suitability for analyzing AsA content variation and suggests that QTL mapping is feasible.

The QTL mapping of AsA content was performed using the previously constructed ‘Ruby-3’ × ‘Kuilv-M’ linkage map. The best linear unbiased estimates (BLUEs) were estimated from phenotypic data collected over the 3 years. One significant QTL was detected on linkage group (LG) 10 at 36 cM with a logarithms of odds (LOD) score of 6.89 (Fig. 1a), which explained 11.46% of the phenotypic variance. The predicted QTL model is QoQo × ooQo with additive gene action. The haplotypes originating from the female parent on homologs 1 and 3 and the male parent on homolog 7 contributed to low AsA content (Fig. 1b). The 1-LOD interval on LG10 was 30–38 cM, corresponding to the physical region Chr10: 4 005 797–5 434 213 in the *A. arguta* genome [40]. A total of 139 genes were identified within the 1-LOD interval and functionally annotated using multiple databases, including GOG, KOG, GO, KEGG, Pfam, Swiss-Prot, eggNOG, and NR (Table S1).

**Figure 1:**
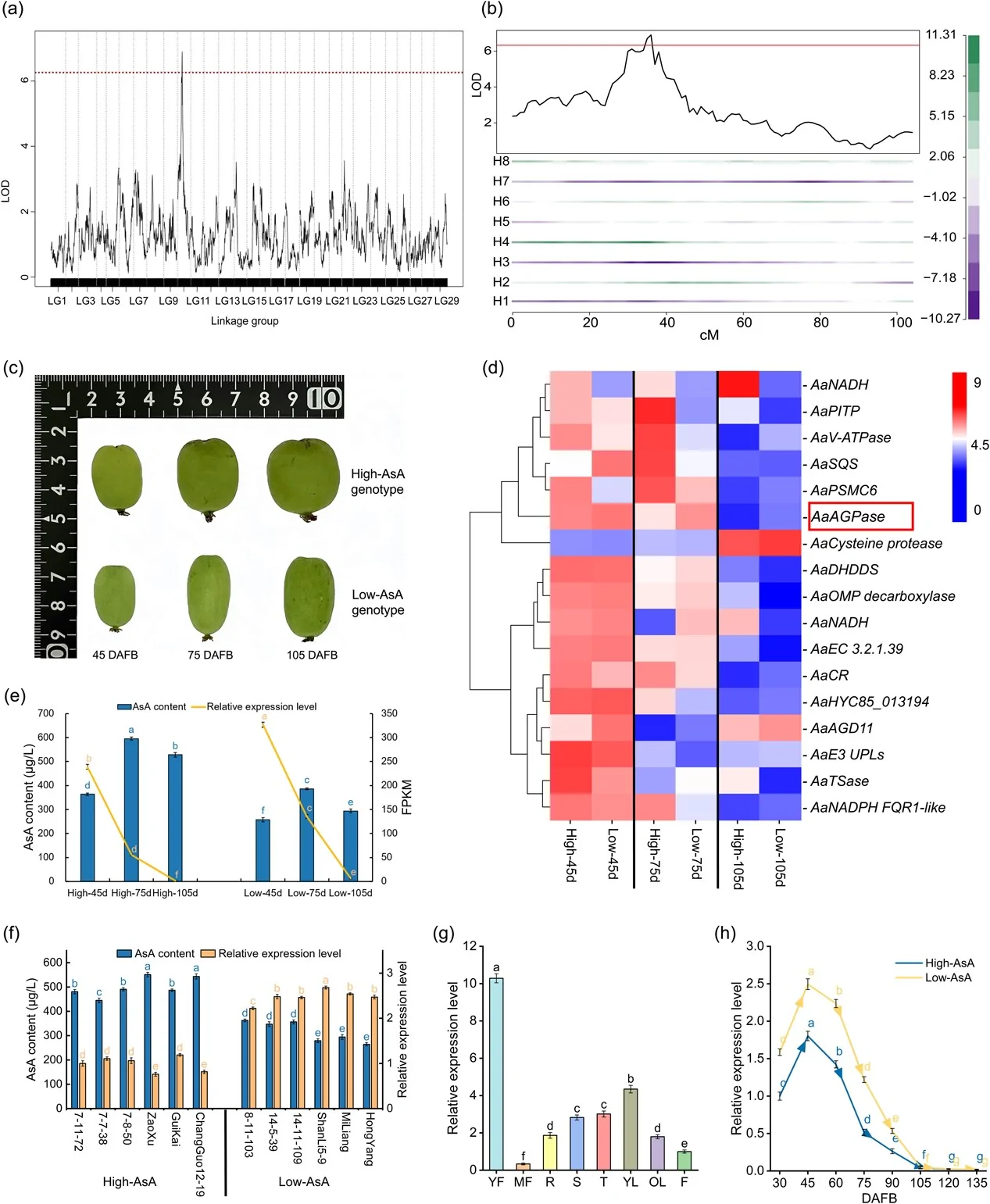
QTL mapping and RNA-seq pinpoints the *AaAGPase* as the candidate responsible for AsA variation. (a) The LOD profiles for QTL scan of the AsA content were plotted across all 29 LGs. (b) Homology analysis of major QTLs for fruit AsA content. The four homologues from the parent ‘Ruby-3’ are labeled as H1–H4 and the other four homologs from the parent ‘Kuilv-M’ are labelled as H5–H8. The reference line indicates the LOD threshold for the QTL calculated based on 1000 permutations. (c) Fruit pictures of RNA-seq samples. (d) Gene expression of *AaAGPase* from17 DEGs between high- and low-AsA genotypes (45, 75 and 105 DAFB stage). (e) The *AaAGPase* expression level and AsA content in RNA-seq samples. (f) The *AaAGPase* expression level and AsA content in the F1 population and kiwifruit germplasm. (g) The tissue-specific expression of *AaAGPase* in kiwifruit. F, flower; MF, mature fruit; OL, old leaves; R, root; S, stem; T, tendril; YF, young fruit; YL, young leaves. Lower-case letters in the column denote the significance level of mean differences at 0.05 (LSD method). (h) The *AaAGPase* expression level throughout fruit developmental stages. Data are presented as mean ± SD (*n* = 3), lower-case letters in the column denote *P* < 0.05.

To identify candidate genes responsible for AsA biosynthesis in kiwifruit, we performed RNA-seq analysis of high- and low-AsA genotypes at three developmental stages (45, 75, and 105 days after the full bloom, DAFB) (Fig. 1c). Eight genes were randomly selected to validate the transcriptome data. The reverse transcription quantitative polymerase chain reaction (RT-qPCR) expression trends were consistent with the RNA-seq results, confirming the reliability of the RNA-seq data (Fig. S1). Transcriptome analysis identified 17 expressed genes within the QTL interval (Fig. 1d), including four differentially expressed genes (DEGs). Among these, *AaAGPase* was downregulated at 45, 75, and 105 days, while *AaTSase*, *AaNADH*, and *AaEC 3.2.1.39* were upregulated at 105 days. Only *AaAGPase* showed significantly differential expression in the high- and low-AsA genotypes throughout fruit development (Fig. 1d), and was thus identified as a candidate gene for further investigation. At the peak of AsA accumulation (75 DAFB), *AaAGPase* expression decreased greatly as the AsA content increased in both genotypes (Fig. 1e). Further analysis of *AaAGPase* expression in the F1 population and kiwifruit germplasm showed that *AaAGPase* was consistently lower in high-AsA genotypes (Fig. 1f).

RT-qPCR analysis of various kiwifruit tissues showed that *AaAGPase* expression was highest in young fruits and lowest in mature fruits. Comparable expression levels were noted in annual branches and tendrils, while flowers exhibited relatively low expression (Fig. 1g). During fruit development, *AaAGPase* expression initially increased, peaking at 45 DAFB, before declining to nearly undetectable levels in mature fruit. Furthermore, *AaAGPase* expression was consistently lower in high-AsA genotypes when compared to low-AsA genotypes (Fig. 1h). These findings indicate that the *AaAGPase* plays a negative regulatory role in AsA biosynthesis in kiwifruit.

### Sequence analysis and promoter activity and subcellular localization of AGPase protein

The full-length coding sequence of *AaAGPase* is 1575 bp, encoding 524 amino acids (aa). Protein sequence alignment across different species showed that *AaAGPase* contained a conserved PLN02241 domain spanning amino acids 92–524 (Fig. 2a and b). To explore the function of *AaAGPase*, we cloned both the *AaAGPase* and its promoter region, which is 2.2 kb in length. Our results indicated that the amino acid sequences of *AaAGPase* are identical in both high- and low-AsA genotypes (Fig. S2). To access the promoter activity of *AaAGPase* in these genotypes, we constructed a reporter gene vector for dual-luciferase (dual-LUC) assay (Fig. 2c). The results showed that the fluorescence from the p*AaAGPase* (low-AsA)::LUC construct were significantly stronger than those from the p*AaAGPase* (high-AsA)::LUC construct. Quantitatively, the relative LUC activity driven by the low-AsA promoter was more than 2-fold greater than that driven by the high-AsA promoter (Fig. 2d).

**Figure 2:**
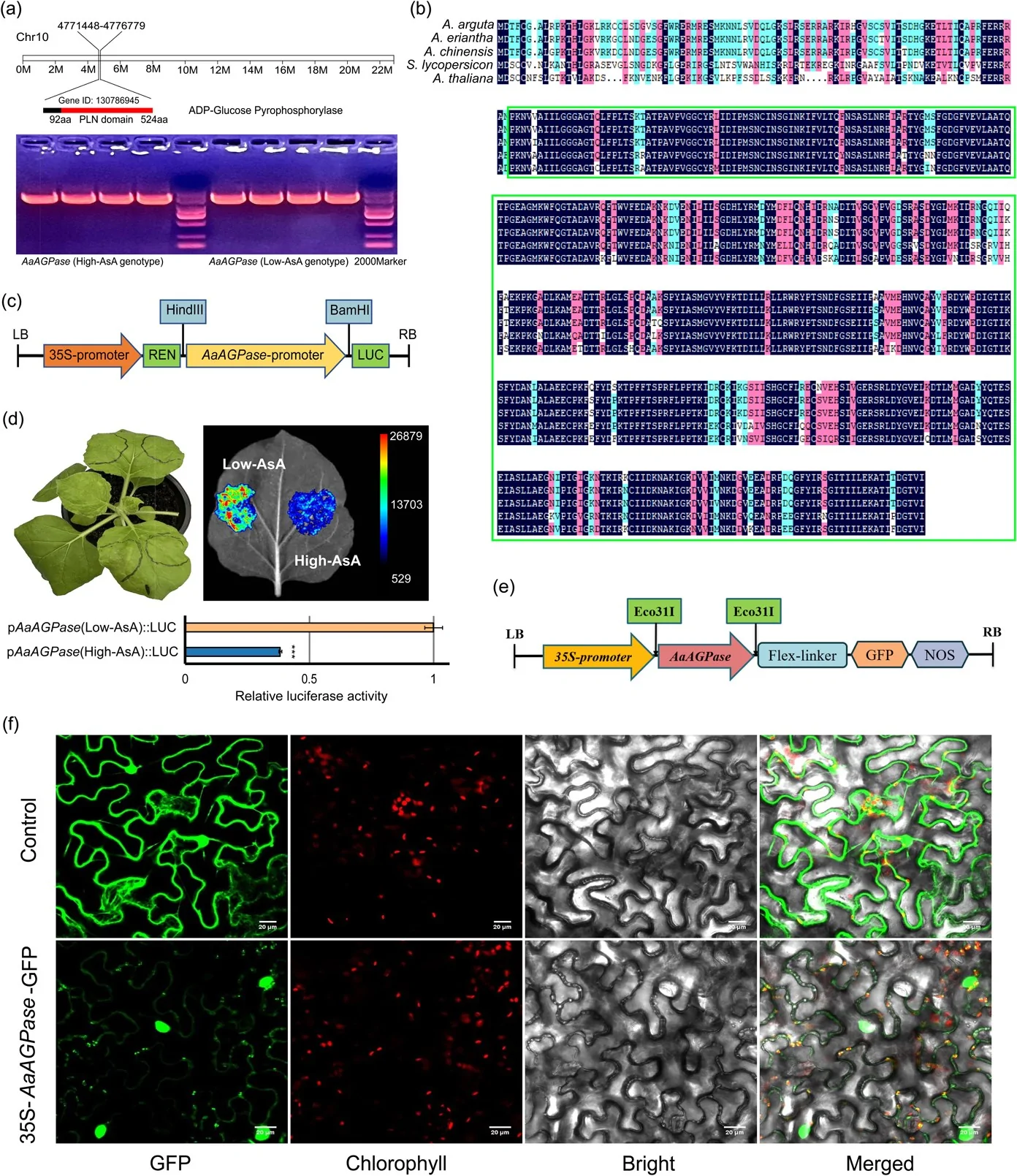
*AaAGPase* sequence analysis, promoter activity analysis, and subcellular localization. (a) Schematic diagram of *AaAGPase* on Chr10. (b) Amino acid sequence alignments of AGPase in kiwifruit, tomato, and *A. thaliana*. The PLN02241 conserved domain is highlighted in box. (c) Schematic representation of dual-LUC reporter gene construct. (d) Fluorescence signal imaging and relative LUC activity in tobacco leaves. The left area was injected with p*AaAGPase*(Low-AsA)::LUC, while the right area was injected with p*AaAGPase*(High-AsA)::LUC. Data are presented as mean ± SD (*n* = 3), Student’s *t*-test, ^***^P < 0.001. (e) Schematic representation of 35S::*AaAGPase*::GFP fusion construct. (f) Subcellular localization of the control and 35S::*AaAGPase*::GFP plasmid in tobacco leaves via confocal microscopy. Bar = 20 μm.

To examine the subcellular localization of AGPase protein, we constructed a pHK-35S-GLosGFP fusion vector (Fig. 2e). The recombinant plasmid was transiently transformed into tobacco leaves under the control of the CaMV 35S promoter. Detection of green fluorescence protein (GFP) confirmed expression of the fusion protein, with fluorescence from *AaAGPase*-GFP primarily localized in the nucleus, and some fluorescence signals were present in the chloroplast, while the GFP signal of the control plasmid was present throughout the cell (Fig. 2f).

### A sequence variation in the *AaAGPase* promoter sequence affects AsA levels

To further understand the function of *AaAGPase*, we compared the sequences of *AaAGPase* promoter (2.2 kb) and its *cis*-acting elements between high- and low-AsA genotype. Our comparative analysis revealed that the promoter sequences of both genotypes exhibited 96.24% similarity and contained several conserved *cis*-acting elements related to stress and defense responses, light responsiveness, and hormone regulation (Table S2). The *AaAGPase* promoter from the low-AsA genotype contained one additional TC-rich element and one fewer CAAT-box (Fig. 3a). Moreover, we identified a polymorphic fragment featuring single nucleotide polymorphisms (SNPs) and insertions-deletions (In-Del), located 367 to 427 bp upstream of the *AaAGPase* start codon (Fig. 3b).

**Figure 3:**
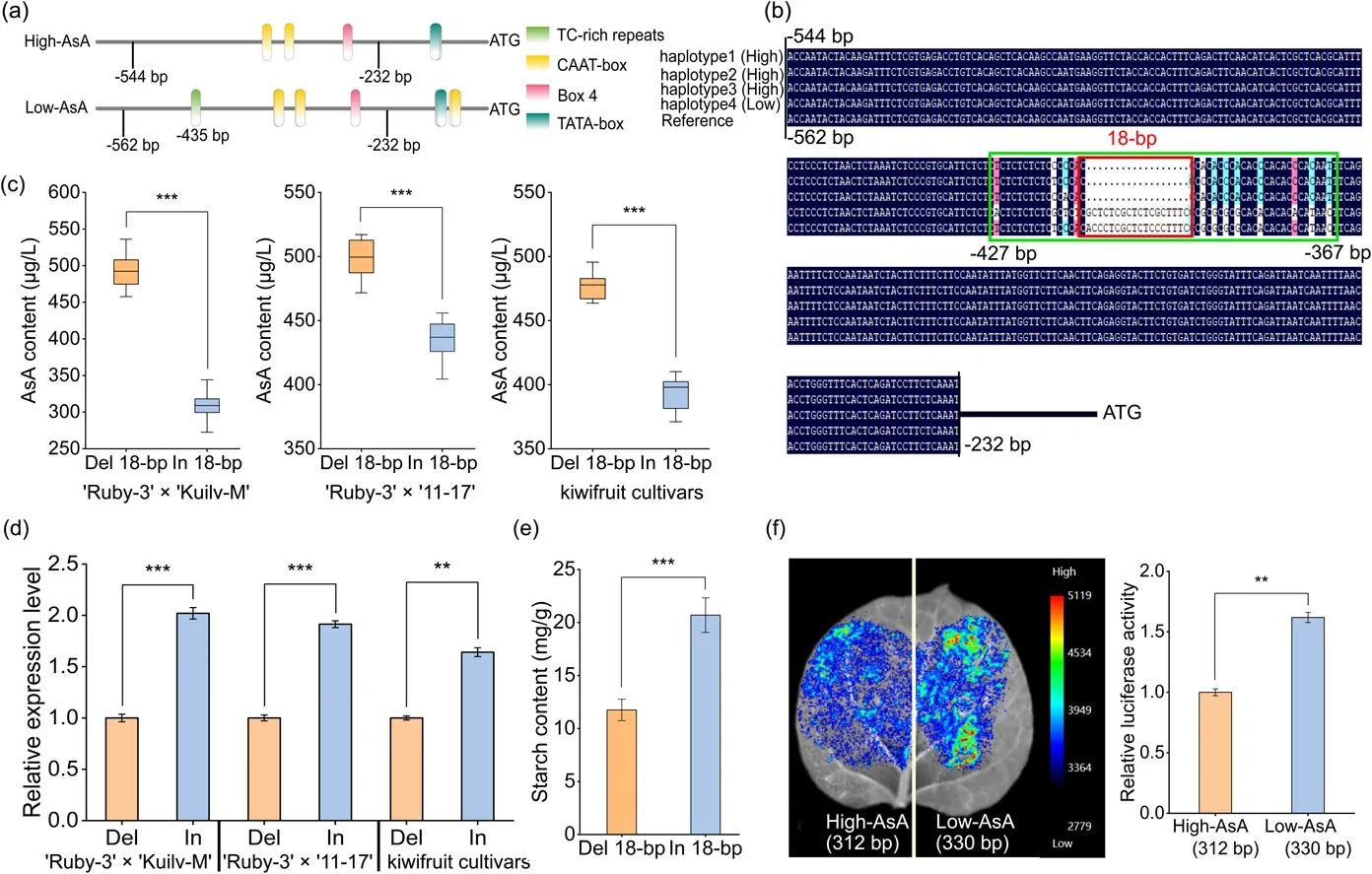
The sequence variation in the *AaAGPase* promoter affects *AaAGPase* expression and AsA levels. (a) Structural differences in the *AaAGPase* promoter between high- and low-AsA genotypes. (b) Sequence alignment of promoter sequences. (c) Genotyping by In-Del marker and AsA level in the ‘Ruby-3’ × ‘Kuilv-M’ population, the ‘Ruby-3’ × ‘11–17’ population, and 88 kiwifruit germplasm accessions. (d) The *AaAGPase* expression levels in high- and low-AsA genotypes from the F1 population and 88 germplasm accessions. (e) Genotyping by In-Del marker and starch level in the ‘Ruby-3’ × ‘Kuilv-M’ population. (f) LUC transient assay for transcriptional activity in high- and low-AsA genotypes using fluorescence imaging and LUC activity in tobacco leaves. Data are presented as mean ± SD (*n* = 3), Student’s *t*-test, ^**^P < 0.01.

We designed specific primers that target the 18-bp polymorphism highlighted in the red box of Fig. 3b. To assess the diagnostic value of this In-Del marker for predicting AsA content, we genotyped and measured the AsA levels of 95 offsprings in the ‘Ruby-3’ × ‘Kuilv-M’ F1 population in 2023. Among them, the average AsA levels of 46 individuals carrying the 18-bp deletion was 308.36 μg/l, significantly lower than the 491.08 μg/l average of the 49 individuals with the 18-bp deletion, representing a 59.26% increase (Fig. 3c). To further test the usefulness of the marker, we genotyped in ‘Ruby-3’ × ‘11–17’ population, the average AsA levels of individuals with 18-bp deletion was 498.66 μg/l, which was significantly higher than that of individuals without deletion (Fig. 3c). In a panel of 88 kiwifruit germplasm accessions, those with the 18-bp deletion had an average AsA levels of 477.09 μg/l, which was significantly higher than those without deletion (Fig. 3c). The RT-qPCR results demonstrated that *AaAGPase* expression was significantly lower in high-AsA genotypes with 18-bp deletion (Fig. 3d). In addition, the starch levels of individuals with 18-bp deletion were significantly lower than those without deletion (Fig. 3e). Correlation analysis showed that AsA levels was significantly negatively correlated with starch levels (*P* < 0.01, *r* = −0.84). Subsequently, we evaluated the effect of this sequence variation on *AaAGPase* transcriptional activity by amplifying this promoter region from both high- and low-AsA genotypes with lengths of 312 and 330 bp, respectively (Fig. 3b). The high-AsA genotype with deletion (312 bp) exhibited a significantly lower fluorescence signal and a lower relative LUC activity (Fig. 3f). These results suggest that the 18-bp deletion reduces the transcriptional activity of the *AaAGPase* promoter, thus affecting both *AaAGPase* expression and AsA levels.

### Overexpression and silencing of *AGPase* affects AsA biosynthesis via the L-galactose pathway

To characterize the function of *AaAGPase* in AsA biosynthesis in kiwifruit, we performed transient expression assays using 35S-driven *AaAGPase* overexpressing and silencing constructs in on-vine *A. arguta* and *Actinidia eriantha* fruits (Fig. S3). OE-*AaAGPase* fruits exhibited more than 250% increase in expression levels and significantly higher AGPase activity compared to controls with the empty vector (EV) (Fig. 4a). However, as D-glucose-1P and D-fructose-6P are substrates for AsA biosynthesis, both levels were found to be downregulated in OE-*AaAGPase* fruits (Fig. 4a). This downregulation was negatively correlated with AGPase activity (*P* < 0.01, *r* = −0.93, −0.94), resulting in a significant decline in AsA levels by more than 40% (Figs 4a and S4). In addition, gene expression analysis of *phosphoglucomutase* (*PGM*) and *glucose-6-phosphoglucose isomerase*, which are downstream of *AaAGPase* in the L-galactose pathway, significantly downregulated in fruits with *AaAGPase* overexpression. The expression levels of enzyme genes downstream of *PGM* and *glucose-6-phosphate isomerase* (*GPI*), such as *GMP*, *GME*, *GGP*, and *GDH*, were also downregulated, while *GPP* levels remained unchanged (Fig. 4b). These findings showed that *AaAGPase* negatively regulated AsA biosynthesis via the L-galactose pathway.

**Figure 4:**
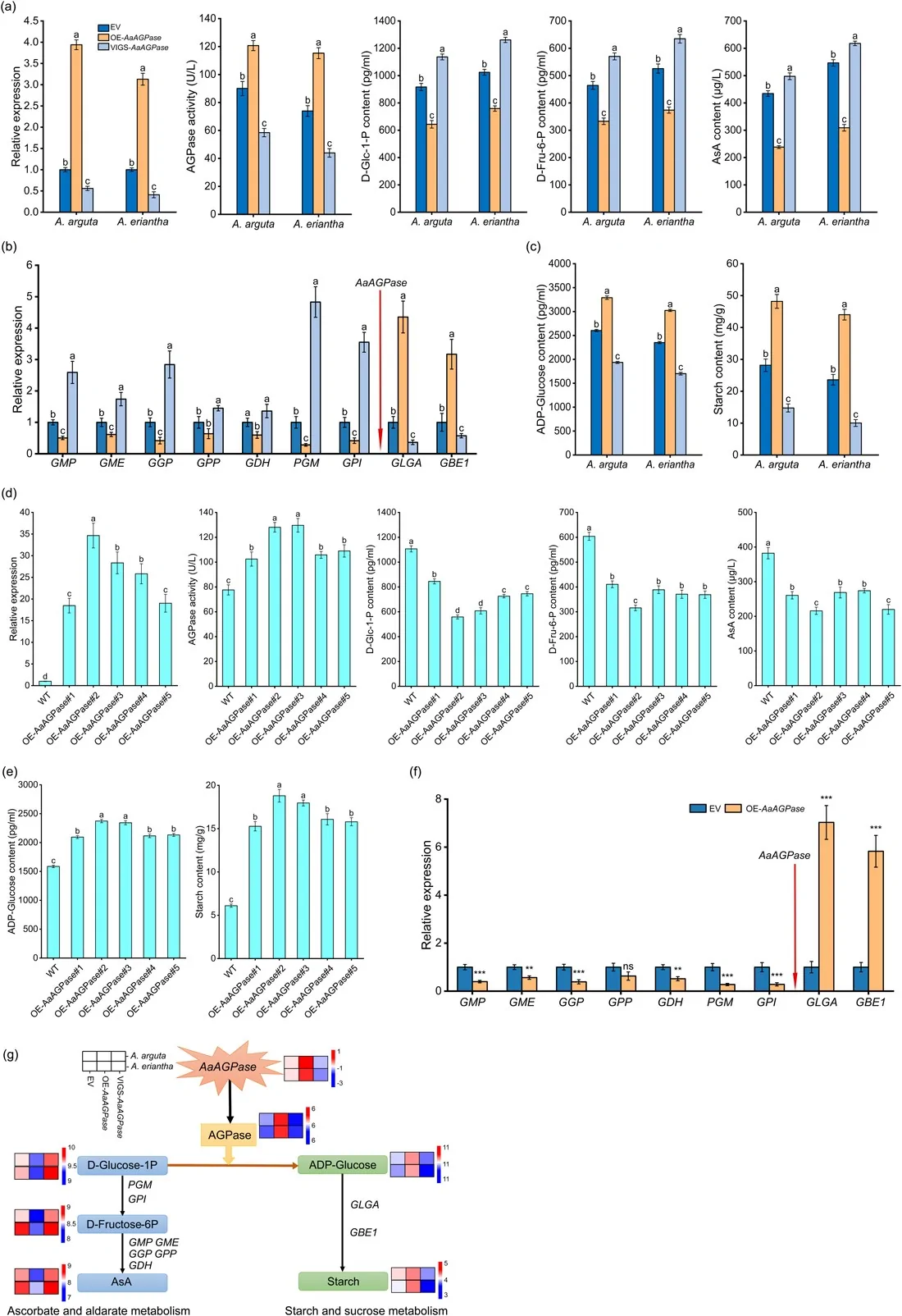
Overexpression or silencing of *AaAGPase* affects AsA levels in transiently transformed fruits and transgenic kiwifruit plants. (a) Levels of *AaAGPase* expression, AGPase activity, D-glucose-1P, D-fructose-6P, and AsA content in transiently transformed fruits. Data are presented as mean ± SD (*n* = 3), relative expression levels were normalized to the kiwifruit *Actin*. (b) Relative expression levels of key genes involved the AsA and starch synthesis pathways in transiently transformed fruits. *GMP*, *GME*, *GGP*, *GPP*, *GDH*, *PGM*, and *GPI* are key genes in the AsA biosynthesis pathway, while *GLGA* and *GBE1* are key genes in the starch synthesis pathway. (c) Levels of ADP-glucose and starch content in transiently transformed fruits. (d) Levels of *AaAGPase* expression, AGPase activity, D-glucose-1P, D-fructose-6P, and AsA content in WT and transgenic kiwifruit plants. (e) Levels of ADP-glucose and starch content in WT and transgenic kiwifruit plants. (f) Relative expression levels of key genes involved the AsA and starch synthesis pathways in transgenic kiwifruit plants. (g) A simplified pathway illustrating AsA and starch metabolism regulated by *AaAGPase*, along with a heatmap showing the metabolites variations detected in transiently transformed fruits.

It is documented that *AaAGPase* catalyzes the conversion of D-glucose-1P to ADP-glucose during starch accumulation. We speculated that *AaAGPase* regulates AsA biosynthesis by competing for the same pool of D-glucose-1P that is used for starch synthesis. To investigate this, we measured the levels of ADP-glucose and starch in OE-*AaAGPase* fruits. Both ADP-glucose and starch were upregulated, exhibiting strong positive correlations with AGPase activity but a negative correlation with AsA content (Figs 4c and S4). Furthermore, the gene expression of key enzymes, *starch synthase* (*GLGA*), and *1,4-α-glucan branching enzyme* (*GBE1*), downstream of *AaAGPase* in the starch metabolic pathway, was significantly upregulated in OE-*AaAGPase* fruits (Fig. 4b).

Virus-induced gene silencing (VIGS) results further support our speculation that VIGS*-AaAGPase* resulted in a reduction of AGPase activity, accompanied by a significant increase in AsA levels, as well as elevated levels of D-glucose-1P and D-fructose-6P compared to the EV (Fig. 4a). Additionally, genes associated with the L-galactose pathway were upregulated (Fig. 4b). In contrast, the levels of ADP-glucose, starch, and genes associated with starch synthesis were found to be downregulated (Fig. 4b and c).

To confirm the role of *AaAGPase* in stable transformation, transgenic kiwifruit plants were generated via *Agrobacterium* transformation. Five independent transgenic plants that overexpressed *AaAGPase* were validated using RT-qPCR (Fig. S5). As expected, overexpression of *AaAGPase* led to a significant decrease in D-glucose-1P, D-fructose-6P, and AsA content, while simultaneously increasing ADP-glucose and starch levels, consistent with the findings from the transient assays (Fig. 4d, e, and  g). RT-qPCR results showed that genes related to the L-galactose pathway were downregulated, while genes related to starch synthesis were upregulated in stable transgenic plants (Fig. 4f). These results confirm that the *AaAGPase* function as a negative regulator in AsA biosynthesis.

### AaGLK acts as a transcriptional activator of *AaAGPase*

In an effort to identify a transcriptional regulator of *AaAGPase*, we analyzed the promoter sequence of *AaAGPase* (Fig. 5a). Multiple transcription factors were screened using the yeast one-hybrid (Y1H) assay (Table S3). This screening revealed that a MYB transcription factor, belonging to the GLK family, designated as AaGLK, binds to the promoter region of *AaAGPase* (Fig. S6). We further investigated the expression levels of *AaGLK* using RNA-seq data, which showed a strong correlation between its expression, *AaAGPase* expression, and AsA levels (Fig. S7).

**Figure 5:**
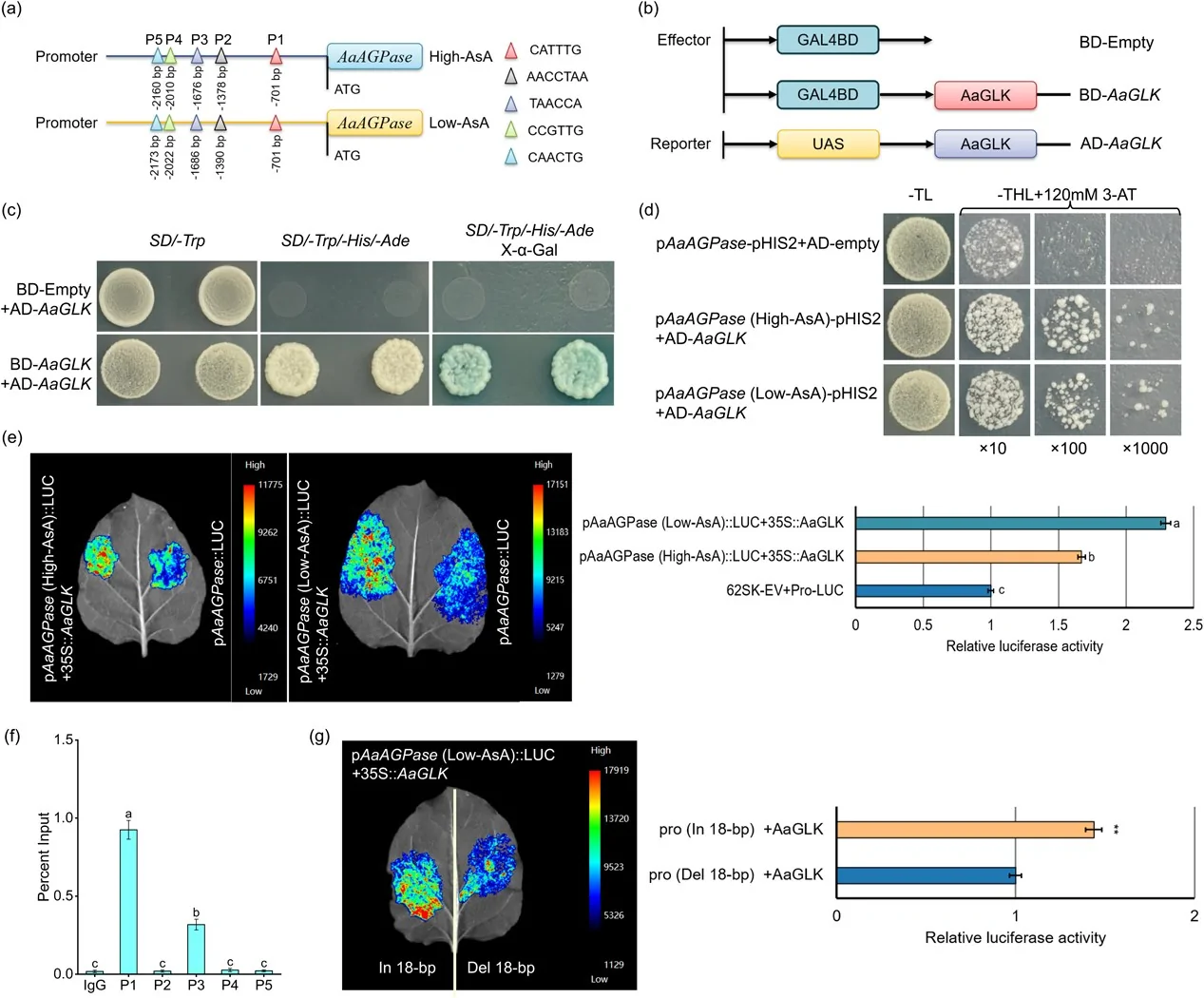
AaGLK activates the expression of *AaAGPase* by binding to its promoter. (a) Schematic diagram illustrates the MYB binding site in the *AaAGPase* promoter. (b) Schematic diagram of the construction of transcription activation test vector. Fusion of AaGLK and GAL4 DNA binding domain of pGBKT7 vector to construct BD-*AaGLK* vector. BD: pGBKT7 vector, AD: pGADT7 vector. (c) AaGLK transcriptional activation in yeast cells. EV pGBKT7 was used as control. (d) Y1H assay shows the interaction between AaGLK (AaGLK::pGADT7) and *AaAGPase* promoter (p*AaAGPase*::pHIS2). The bait–prey combination: p*AaAGPase*-pHIS2 (high- and low-AsA) + *AaGLK*-pGADT7, the negative control combination: p*AaAGPase*-pHIS2 + pGADT7 EV. (e) LUC assay measures the transcriptional activity of AaGLK between high- and low-AsA genotypes of *AaAGPase* using fluorescence signal imaging and relative LUC activity in tobacco leaves. The left area was injected with 35S::*AaGLK* and p*AaAGPase*(high- and low-AsA)::LUC, and the right area was injected with the pBI121 EV and p*AaAGPase*::LUC. (f) CUT&RUN-qPCR verification of AaGLK binding to the *AaAGPase* promoter in kiwifruit leaves expressing *AaGLK* with a GFP tag. The percent input method was selected for quantification via qPCR. IgG represents the negative control. Data are presented as mean ± SD (*n* = 3), lower-case letters in the column denote *P* < 0.05. (g) LUC assay measures the transcriptional activity of the GLK gene using the *AaAGPase* promoter sequence, with and without an 18-bp deletion using fluorescence signal imaging and relative LUC activity in tobacco leaves. The left area was injected with 35S::*AaGLK* + p*AaAGPase* (low-AsA) (in 18 bp)::LUC, and the right area was injected with 35S::*AaGLK* + p*AaAGPase* (low-AsA) (Del 18-bp)::LUC. Data are presented as mean ± SD (*n* = 3), Student’s *t*-test, ^**^P < 0.01.

Sequence analysis revealed that the protein sequences of AaGLK were identical in both high- and low-AsA genotypes (Fig. S8). To examine the transcriptional activation of the AaGLK protein, we fused the full-length coding DNA sequence (CDS) of *AaGLK* with the GAL4 DNA-binding domain of pGBKT7 (Fig. 5b). As shown in Fig. 5c, the colonies turned blue on selective medium containing X-α-Gal and grew on SD/−Trp/−His/−Ade plates, indicating that the AaGLK protein activates downstream reporter gene expression and functions as a transcriptional regulator of structural genes. Compared to yeast cells harboring the empty pGADT7 vector, those co-expressing p*AaAGPase*::pHIS2 + AaGLK::pGADT7 exhibited normal growth on selective screening medium (Fig. 5d). Moreover, a dual-LUC assay was performed to further explore whether AaGLK regulates the promoter activity of *AaAGPase*. Infiltration with 35S:*AaGLK* resulted in a significant increase in both relative LUC activity and fluorescence signal in tobacco leaves, compared to the EV (Fig. 5e). This indicates that AaGLK binds and activates the *AaAGPase* promoter for both high- and low-AsA genotypes. However, the low-AsA genotype of *AaAGPase* promoter showed stronger transcriptional activation of AaGLK than the high-AsA genotype, as indicated by higher relative LUC activity and fluorescence of p*AaAGPase* (low-AsA)::LUC + 35S::*AaGLK* (Fig. 5e). To verify the interaction between AaGLK and the *AaAGPase* promoter *in vivo*, CUT&RUN-qPCR was carried out using cell extracts from kiwifruit leaves transiently expressing AaGLK-GFP fusions. Five AaGLK-binding regions in the *AaAGPase* promoter were tested (Fig. 5a), with specific enrichment detected at P1 and P3 (Fig. 5f). The stronger signal at P1 suggests this region serves as the primary binding site.

Previously, we demonstrated that the 18-bp deletion in the promoter of *AaAGPase* was associated with low *AaAGPase* expression (Fig. 3d). To investigate whether this 18-bp deletion affects the transcriptional activation of *AaAGPase* by AaGLK, we deleted the 18-bp fragment from the low-AsA genotype of *AaAGPase* promoter to compare the transcriptional activation between this modified and the intact promoter sequence. LUC assays showed that the modified promoter with 18-bp deletion resulted in a decrease in fluorescence signal and relative LUC activity, indicating reduced activation of the *AaAGPase* by AaGLK (Fig. 5g).

### AaGLK negatively regulates AsA biosynthesis via AGPase

To examine the regulatory effects of AaGLK on the AsA biosynthesis, we performed transient overexpression and silencing experiments in *A. arguta* fruits and transgenic kiwifruit plants with overexpressing *AaGLK*. Compared with EV, transient overexpression of *AaGLK* led to a 66% increase in the *AaAGPase* expression and a 27% reduction in AsA levels. Silencing *AaGLK* by VIGS reversed the phenotype associated with overexpression, resulting in an upregulation of AsA levels and a downregulation of starch levels. To investigate whether the effects of *AaGLK* on AsA and starch levels were mediated through *AaAGPase*, we co-expressed *AaGLK* and *AaAGPase* in the OE-*AaGLK* + OE-*AaAGPase*, which resulted in AsA levels being downregulated to the same level as observed in the VIGS-*AaGLK* + OE-*AaAGPase*. The fruit exhibited characteristics similar to those seen when only *AaAGPase* was manipulated. A similar relationship was observed in starch levels, where starch levels were upregulated in the OE-*AaGLK*, while the co-expression of *AaGLK* and *AaAGPase* produced starch levels in the OE-*AaGLK* + OE-*AaAGPase* comparable to those in the VIGS-*AaGLK* + OE-*AaAGPase* group. Silencing both *AaGLK* and *AaAGPase* or VIGS-*AaGLK* + OE-*AaAGPase* approach reversed the phenotype associated with overexpression (Fig. 6a–d). This shows that the AaGLK-AaAGPase module functions as a shared transcriptional relay for the coordinated regulation of both AsA and starch levels, with *AaAGPase* being the dominant factor regulating metabolic output, activated by AaGLK.

**Figure 6:**
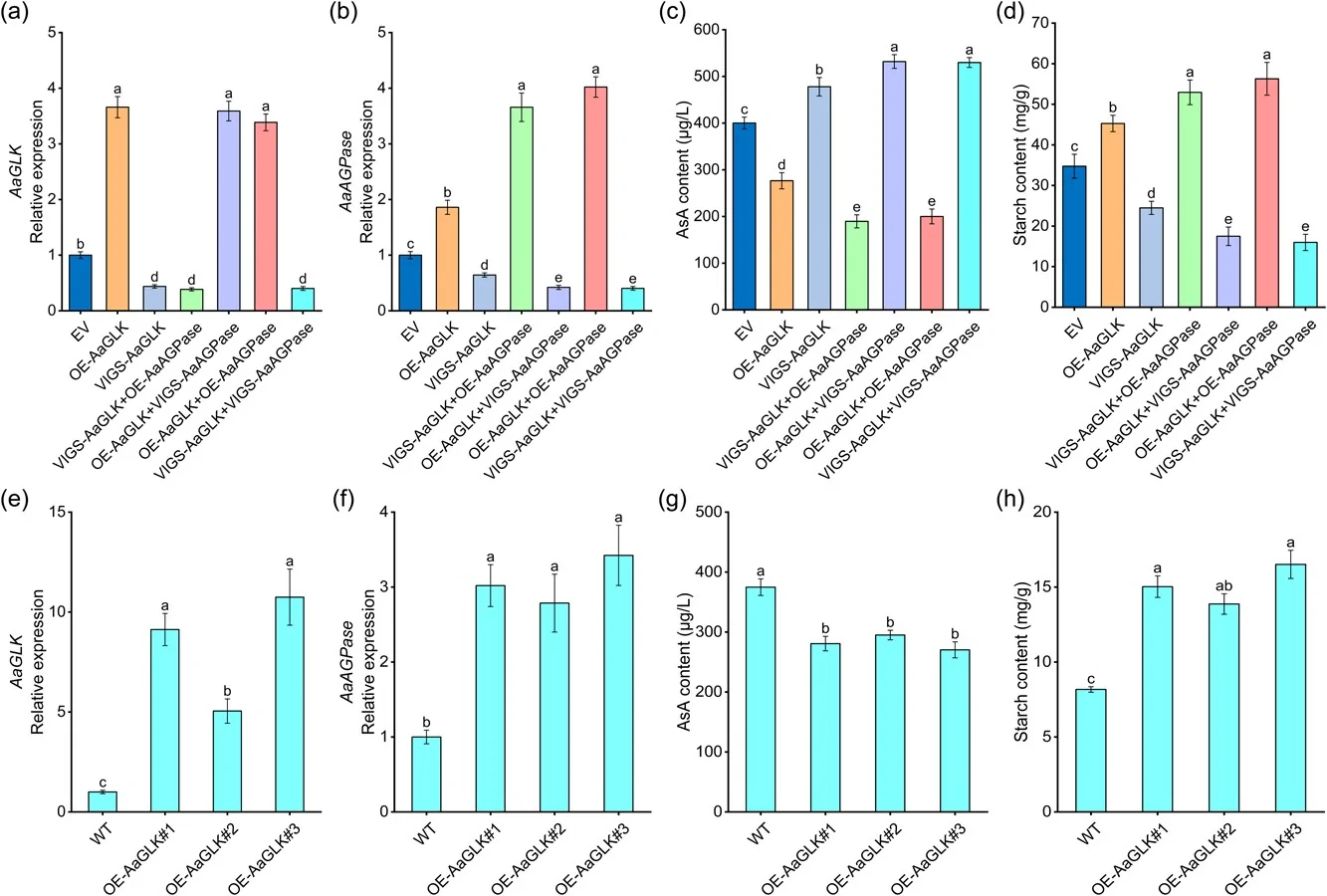
Overexpression or silencing of *AaGLK* affects AsA and starch levels in transiently transformed fruits and transgenic kiwifruit plants. Relative expression levels of *AaGLK* (a) and *AaAGPase* (b), and the levels of AsA (c) and starch (d) in transiently expressed fruits. Relative expression levels of *AaGLK* (e) and *AaAGPase* (f) in transgenic kiwifruit lines overexpressing *AaGLK*. Relative expression levels were normalized to the kiwifruit *Actin*. Levels of AsA levels (g) and starch levels (h) in transgenic kiwifruit plants overexpressing *AaGLK*. Data are presented as mean ± SD (*n* = 3), lower-case letters in the column denote *P* < 0.05.

Three independent transgenic plants that overexpressed *AaGLK* were generated and validated through RT-qPCR (Figs 6e and S5). These transgenic plants exhibited a significant upregulation of *AaAGPase* expression (Fig. 6f). Consistent with the transient overexpression assays, the AsA levels were downregulated in these OE-*AaGLK* plants compared to wild-type (WT) plants, while starch levels were significantly upregulated (Fig. 6g and h).

## Discussion

Since humans cannot synthesize AsA, dietary intake from vegetables and fruits is essential. Kiwifruit accumulates AsA at concentrations up to 10 times higher than those found in apples and pears, making it an excellent dietary source and an important target for breeding. The AsA biosynthesis in kiwifruit is primarily mediated by the L-galactose pathway, which is driven by a series of core enzymatic reactions. The biochemical pathways underlying AsA accumulation in kiwifruit have been well characterized; however, little is known about the genetic determinants and regulatory mechanisms governing these processes. In this study, we genetically mapped a locus controlling AsA levels and identified a promoter sequence variation in the *AGPase* that affects AsA levels. Moreover, we elucidated the pivotal role of the *AGPase* in determining AsA levels, likely through its control of starch substrates homeostasis. Our findings suggest an expanded functional scope for *AGPase* in fruit development and point to a novel regulatory network that integrates AsA and starch biosynthesis.

Our study identified a QTL on chr10 affecting AsA levels in a tetraploid *A. arguta* biparental population. This QTL explains 11.46% of the phenotypic variance, indicating that the genetic architecture likely involves additional contributing loci. In contrast, analysis of *A. eriantha* × *Actinidia chinensis* backcross populations revealed a major supergene locus, qAsA26.1, on chr 26. This locus significantly increases AsA content and points to a clustered assembly of functionally related genes [41]. Complementing these findings, a genome wide association study in *A. eriantha* independently implicates multiple loci for AsA variation [42]. Together, these results provide convergent evidence for the polygenic nature of AsA accumulation across *Actinidia* species.

Through differential expression analysis of genes expressed within the QTL, we pinpointed a candidate gene, *AaAGPase*, which is well-known to convert D-glucose-1P and ATP into ADP-glucose for starch synthesis. Consistent with its established role in starch metabolism, we found that *AaAGPase* was highly expressed during early fruit development. This finding aligns with previous studies that reported high *AaAGPase* expression during cell division and expansion in *Actinidia deliciosa* kiwifruit [43]. Furthermore, differential expression of the *AaAGPase* between high- and low-AsA genotypes was associated with a sequence variation in its promoter region, which ultimately affected AsA levels. We integrate these genetic and metabolic findings into a proposed model where promoter variation in the *AaAGPase* acts as a switch directing carbohydrate substrates between starch and AsA biosynthesis in fruit, as shown in Fig. 7. Supporting this hypothesis, substrates measurements showed that overexpression of *AaAGPase* resulted in significantly lower levels of D-glucose-1P and AsA, while simultaneously increasing levels of ADP-glucose and starch. Additionally, overexpression of *AaAGPase* downregulated genes involved in the L-galactose pathway, while upregulated the genes involved in the starch synthesis pathway (Fig. 4b and f). Similar associations between competing carbohydrate metabolic pathway expression and fruit AsA levels have been reported in studies of tomato interspecific introgressions and ripening [44, 45].

**Figure 7:**
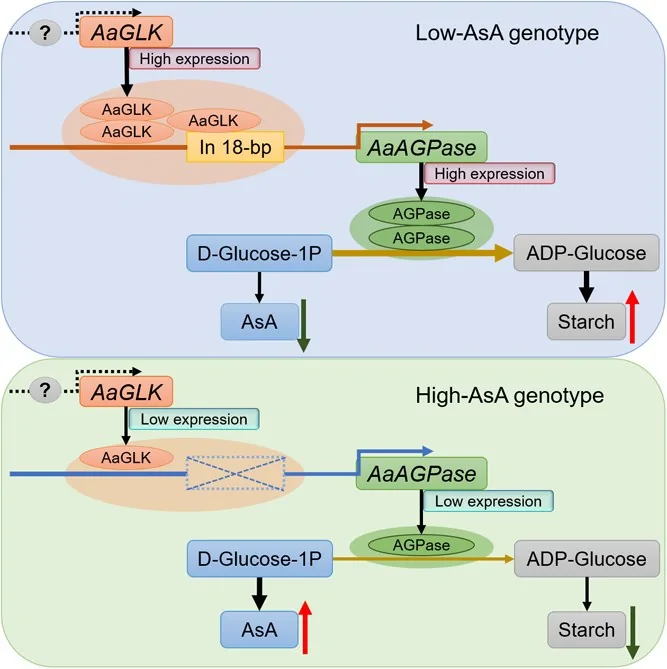
The proposed model for the role of AaGLK-*AaAGPase* module in the AsA synthesis in kiwifruit. In the low-AsA genotype, the higher expression of *AaGLK*, along with the presence of an 18-bp sequence in the promoter of *AaAGPase*, enhances the activation of *AaAGPase* transcription. This stronger activation significantly increases *AaAGPase* expression, which in turn increases its enzymatic activity. As a result, more D-glucose-1P is directed into starch synthesis, depleting the substrate pool available for AsA biosynthesis. Conversely, the high-AsA genotype shows lower *AaGLK* expression and its sequence variation particularly the 18-bp deletion in *AaAGPase* promoter. This variation reduces AaGLK-dependent transcriptional activation, leading to decreased *AaAGPase* expression. With lower AGPase activity, less D-glucose-1P is diverted toward starch synthesis, making more precursor available and ultimately increasing AsA level.

Transcription factors are master regulators upstream of *AGPase*, directly controlling its expression to govern starch biosynthesis. For instance, the CmMYB44 represses *AGPase* expression by binding to its promoter, leading to reduced starch accumulation and affecting the flavor of oriental melon fruit [46]. In Arabidopsis, the sugar-responsive transcription factor MYBS1 activates *AGPase* transcription by binding a specific TA-box element [47]. In our study, using Y1H, CUT&RUN-qPCR, and LUC assays, we identified a Golden 2-like transcription factor, AaGLK, that binds to and activates the *AaAGPase* promoter. Furthermore, we discovered a sequence variation within this promoter. This variation resulted in reduced transcriptional efficiency and explained the differential *AaAGPase* expression observed between genotypes. GLK/G2-like transcription factors are conserved MYB-GARP regulators that control chloroplast biogenesis and photosynthesis across plant species, with additional roles in development, metabolite synthesis, and stress responses that ultimately influence yield and carbon allocation [48, 49]. In tomato, the GLK2/UNIFORM ripening mutation disrupts SlGLK2, resulting in pale green fruit due to a reduction in chlorophyll and chloroplast numbers. Overexpression of GLK2 has been shown to restore fruit color and enhance carbohydrate levels in ripe tomatoes [50, 51]. GLK transcription factors drive the expression of photosynthesis-associated genes by binding to the promoters of these genes [38]. Comparative analysis of GLK transcription factor binding across species reveals that while GLK functions are generally conserved, most binding sites are species-specific, often near photosynthetic genes dependent on GLK [52]. Previous study has identified a GLK homolog in *A. chinensis* kiwifruit, AchGLK, with high expression in mature leaves and fruits 60 days post-anthesis. Overexpression of *AchGLK* in transgenic tomato produced dark green immature fruit with increased chlorophyll, larger chloroplasts, and higher levels of carotenoids and sugars in ripe fruit [39]. Our findings showed direct evidence that GLK plays a regulatory role in sugar and starch mechanisms via transcriptional activation of *AGPase*. Furthermore, our experiments showed that overexpression and silencing of *AaGLK* resulted in variations in AsA levels via regulation of *AaAGPase* expression. In Arabidopsis, GLKs act downstream of HY5, integrating light signals to co-regulate photosynthetic gene expression [53]. In tomato, *SlGLK2* expression is modulated by light and auxin, indicating a connection to the network of sugar metabolism enzymes [54]. There is also evidence from kiwifruit suggesting light-induced AsA biosynthesis [55]. Therefore, we propose that the AaGLK-AaAGPase module may be light dependent, consequently affecting AsA levels. This presents a valuable area for future research.

Our molecular characterization of the AaGLK-AaAGPase module highlights that understanding the genetic architecture of AsA biosynthesis is critical for developing molecular breeding strategies aimed at enhancing fruit AsA levels. Molecular markers are characterized by high levels of polymorphism and are generally unaffected by environmental factors, such as climate and cultivation conditions. They can be detected in various tissues, organs, and developmental stages of plants, making them powerful tools for genetic analysis. MAS is particularly advantageous for perennial and dioecious species, such as kiwifruit, where the evaluation of phenotypes requires multiple years and substantial resources. In kiwifruit breeding programs, MAS not only accelerate the breeding cycle but also improve selection accuracy and reduce the costs associated with long-term maintenance of breeding populations [56]. The In-Del marker developed in this study are useful for metabolically informed breeding. Given that kiwifruit plants typically take 3–6 years to bear fruits, genotyping for this regulatory locus will allow us to predict the metabolic tendencies of seedlings, enabling informed decisions for early selection.

In conclusion, our study addresses the question of whether major genetic determinants for AsA exist within cultivated kiwifruit. By integrating genetic mapping and functional analysis, we identified a novel QTL and elucidated its molecular mechanism through the differential expression of the *AaAGPase* activated by AaGLK due to promoter variation. This work significantly expands the regulatory function of *AGPase* in AsA biosynthesis, which is governed by an integrated network characterized by metabolic crosstalk, where a major regulator *AGPase* can be directly targeted for trait improvement. Thus, our work enables MAS for enhanced nutritional quality, offering a clear pathway from gene discovery to cultivar development.

## Materials and methods

### Plant materials

The F1 population was generated by crossing ‘Ruby-3’ (*A. arguta*, tetraploid, female) and ‘KuiLv-M’ (*A. arguta*, tetraploid, male). ‘Ruby-3’ was collected from central China, with an AsA content of 360 μg/l, while ‘KuiLv-M’ was from Northeast China, and its AsA content is unknown since male kiwifruit plants do not produce fruit. The F1 seedlings were planted in 2018, and 261 female individuals were phenotyped for AsA content, with 20 mature fruits (120 DAFB) harvested from each year. The fruits samples were cut, frozen, and stored at −80°C for AsA content determination.

For RNA-seq, two F1 individuals (8-2-12 and 9-5-47) were selected from the ‘Ruby-3’ × ‘KuiLv-M’ population representing high- and low-AsA genotypes were sampled at three stages: 45 DAFB as pre-AsA accumulation stage, 75 DAFB as AsA peak stage, and 105 DAFB as AsA stable stage). The RT-qPCR validation used the same RNA samples as RNA-seq analysis. For transient expression assays, the 7-7-38 (*A. arguta*) and ‘Zaoxu’ (*A. eriantha*) were used to verify gene function.

To validate candidate gene expression, six genotypes from the ‘Ruby-3’ × ‘KuiLv-M’ population (three with high AsA: 7-7-38, 7-8-50, and 7-11-72; three with low AsA: 8-11-103, 14-5-39, and 14-11-109) and six kiwifruit accessions (‘Zaoxu’, ‘Guikai’, ‘Changguo 12–19’, ‘Shanli 5–9’, ‘Miliang’, and ‘Hongyang’) were selected. Tissue-specific expression was assessed using roots, stems, tendrils, young leaves, old leaves, flowers, and fruits at two stages collected in 2025. Expression trends during fruit development were analyzed from fruit sampled at eight time points (30, 45, 60, 75, 90, 105, 120, and 135 DAFB).

In addition, a tetraploid *A. arguta* ‘Ruby-3’ × ‘11–17’ population and a panel of 88 kiwifruit germplasm were used to develop the marker related to AsA content. The 88 accessions of kiwifruit germplasm included *A. chinensis*, *A. arguta*, *A. eriantha*, *A. deliciosa*, *Actinidia valvata*, *Actinidia macrosperma*, *Actinidia longicarpa*, *Actinidia polygama*, *Actinidia kolomikta*, and *Actinidia melanandra*. All plant materials were maintained under standard management in Zhengzhou Fruit Research Institute, China.

### Determination of AsA, AGPase activity, D-glucose-1P, D-fructose-6P ADP-glucose, and starch

AsA content was determined using an enzyme-linked immunosorbent assay (ELISA) kit (Jiangsu Meimian Industrial Co., Ltd, Jiangsu, China) according to the manufacturer’s protocol [57–59]. Briefly, antibody–antigen–enzyme-labeled antibody complexes were prepared by double antibody sandwich method. After washing, HRP-conjugate reagent was added and incubated at 37°C for 30 min, followed by the addition of chromogen solutions A and B, and another incubation at 37°C for 10 min. The stop solution changed the color from blue to yellow, with intensity correlating to AsA content. Absorbance at 450 nm was measured using a microplate reader within 15 min and AsA content was calculated from standard curves. Similarly, the AGPase activity and the levels of D-glucose-1P, D-fructose-6P, and ADP-glucose were determined using ELISA kits [57–59]. Starch content was quantified using a plant starch detection kit (Jiangsu Addison Biotechnology Co., Ltd, Jiangsu, China). Briefly, starch was decomposed into glucose via hydrolysis, with glucose quantification determined by anthrone colorimetry at a 620 nm. Each metabolite was measured in three biological replicates.

### Quantitative trait locus analysis and candidate gene prediction

The ‘Ruby-3’ × ‘Kuilv-M’ population was genotyped using the kiwifruit 135 K SNP array, and an integrated linkage map based on SNP markers was constructed for the population [60]. BLUEs of AsA content for individuals from 2021 to 2023 were calculated using a linear mixed model. Genotype probabilities of the population were estimated allowing for multivalent pairing during parental meiosis and QTL analysis of the trait was performed using the R package polyqtlR [61]. Significance thresholds for LOD were determined through 1000 permutations at *α* = 0.05. The LOD profile plots across all chromosomes and the QTL model configurations were generated by polyqtlR.

### Transcriptome analysis and RT-qPCR validation

Total RNA was extracted using the RNAprep Pure Plant Kit (Tiangen, Beijing, China). Sequencing libraries were generated using Hieff NGS Ultima Dual-mode mRNA Library Prep Kit for Illumina (Yeasen Biotechnology Co., Ltd, Shanghai, China). The library fragments were purified with AMPure XP system (Beckman Coulter, Beverly, USA). The libraries were sequenced on an Illumina NovaSeq platform to generate 150-bp paired-end reads. The clean reads were mapped to the reference genome using Hisat2. Gene expression levels were expressed as fragments per kilobase of transcript per million mapped reads (FPKM) [62]. The DEGs were identified by using DESeq2 (v1.22.2) [63] [|log2(Fold Change)| ≥ 2 and the false discovery rate < 0.01].

RT-qPCR was performed using the NovoStart®SYBR qPCR SuperMix Plus Kit (Novoprotein, Suzhou, China) on a Roche Light 480 System (Roche, Basle, USA). The Primer premier 5.0 tool was used to design primers based on the CDS of each gene. Relative expression of candidate genes was calculated by the 2^–ΔΔCT^ method [64], and the kiwifruit *Actin* was used as the internal reference gene to normalize the results. Gene expression analysis was performed in four technical replicates and three biological replicates. Details of the primers utilized are provided in Table S4.

### Cloning and analysis of gene sequences

The full-length coding sequence of *AaAGPase* and *AaGLK* and the promoter sequence (2.2 kb) of *AaAGPase* were cloned based on the *A. arguta* reference genome [40]. The promoter sequence of *AaAGPase* was analyzed with the PlantCARE database [65]. Multiple sequences were aligned by DNAMAN (v9.0.1.116). The primers used are provided in Table S4.

### Dual-luciferase reporter assays

To verify promoter activity, the 2.2-kb promoter fragments of the *AaAGPase* and molecular marker fragment (312 and 330 bp) were cloned into the pGreenII0800-LUC vector [66]. *Agrobacterium tumefaciens* strain GV3101 cells were used for transformation and the suspension was infiltrated into tobacco (*Nicotiana benthamiana*) leaves. After 48 h at 25°C, fluorescence was observed using a fluorescence imager and relative LUC activity (F-LUC/R-LUC) was detected using a fluorescence detection kit (Vazyme, Nanjing, China).

For interaction verification, the *AaAGPase* promoter sequences from both high- and low-AsA genotypes were inserted into the pGreenII 0800-LUC vector, while the AaGLK CDS was cloned into the pBI121 vector. Subsequently, the reporter plasmid was transformed into *A. tumefaciens* competent cells GV3101 (*pSoup*), while the effector plasmid was transformed into GV3101 competent cells. The mixture was infiltrated into tobacco leaves at a 1:3 volume ratio. The combination of p*AaAGPase*-pGreenII 0800-LUC + pBI121-*AaGLK* was defined as the experimental group, while p*AaAGPase*-pGreenII 0800-LUC + pBI121-EV served as the negative control group.

### Subcellular localization

The CDS of *AaAGPase*, excluding the stop codon, was inserted into the pHK-35S-GLosGFP vector using the Eco31I restriction site. The plasmids were introduced into tobacco leaves via *A. tumefaciens* GV3101-mediated infiltration. After 2 days in low light, GFP fluorescence was visualized using confocal microscopy.

### Transient expression assay and transgenic kiwifruit plants

The CDS of *AaAGPase* and *AaGLK* were cloned into pBI121 and transformed into *A. tumefaciens* strain GV3101 cells. When *AaGLK* and *AaAGPase* were co-overexpressed, *Agrobacterium* strains containing *AaGLK* and *AaAGPase* were co-infiltrated (1:1) into kiwifruit fruits. For VIGS, 210-bp fragments corresponding to each gene were cloned into the pTRV2 and transformed into *A. tumefaciens* strain GV3101 cells. The suspension (0.4 ml, OD_600_ = 0.6) was injected into the kiwifruit fruits. The fruits after infiltration were bagged for 24 h and then transferred to normal growth conditions. Samples were collected on Day 10 for RT-qPCR and metabolite analysis.

For generation of stable transgenic lines, the pBI121-*AaAGPase* and pBI121-*AaGLK* plasmids were transformed into the kiwifruit calli (*A. chinensis* cv. Hongyang) [67]. The transgenic plants were validated by RT-PCR. The primers used for vector construction and identification of transgenic lines are listed in Table S4.

### Transcriptional activation analysis

The full-length cDNA of *AaGLK* were fused into pGBKT7 vector to generate the BD-AaGLK construct, which was then transformed into yeast strain AH109. Monoclonal colonies were picked from SD/−Trp selective plates and resuspended in 0.9% normal saline. Positive clones were identified by PCR, inoculated in liquid SD/−Trp medium and cultured until an OD_600_ of 0.2. Subsequently, 10 μl of the diluted bacterial culture was spotted onto three types of selective media: SD/−Trp, SD/−Trp/-His/−Ade, and SD/−Trp/−His/−Ade + X-α-gal. All spotted plates were incubated at 30°C for 3–5 days to observe growth and color development. The primers used are provided in Table S4.

### Yeast one-hybrid assay

The *AaAGPase* promoter fragment was cloned into pHIS2 to generate the bait vector p*AaAGPase*-pHIS2. The CDS of *AaGLK* was cloned into the pGADT7 to generate the prey vector *AaGLK*-pGADT7. Two sets of plasmids were separately co-transformed into Y187 yeast competent cells: (i) the bait–prey combination (p*AaAGPase*-pHIS2 + *AaGLK*-pGADT7) and (ii) the negative control combination (p*AaAGPase*-pHIS2 + empty pGADT7 vector). The yeast cells were spread onto SD/−Trp/−Leu selective plates and incubated at 30°C for 3–5 days. The bacterial suspensions were adjusted to an OD_600_ of 0.2 using 0.9% NaCl solution, followed by serial dilutions to 10×, 100×, and 1000×. A 10 μl aliquot of each diluted bacterial suspension was spotted onto SD/−Trp/−Leu (basic selection medium) and SD/−Trp/−Leu/−His + 3-AT (stringent selection medium for verifying protein-DNA binding). All spotted plates were incubated at 30°C for 3–5 days, after which yeast colony growth was observed and recorded. The primers used are provided in Table S4.

### CUT&RUN-qPCR

The CDS of *AaGLK* were cloned into pBI121-GFP and transformed into *A. tumefaciens* strain EHA105 cells. Kiwifruit leaves transiently overexpressing AaGLK-GFP fusions were used for CUT&RUN. Cells were subjected to CUT&RUN assay following the protocol described by Skene and Henikoff with minor modifications [68, 69]. CUT&RUN-qPCR was performed using the Hyperactive pA/G-MNase CUT&RUN assay Kit (HD103, Vazyme Biotech Co., Ltd) according to the manufacturer’s instructions. Immunoglobulin G (IgG) was used as a negative control. The primers used are provided in Table S4.

### Statistical analysis

Statistical significance between groups was assessed using independent sample *t*-tests. Results are presented with ^*^P < 0.05, ^**^P < 0.01, and ^***^P < 0.001, and different letters denote significance (*P* < 0.05) as determined by analysis of variance (ANOVA). Data are expressed as mean ± SE. Visualizations were done using R4.1.0 and Origin 2024 software.

## Supplementary Material

Web_Material_uhag212

## Data Availability

Data that support the findings of this study are available within the paper and its supporting information files. Raw data and materials are available upon request.
